# Supplementary material for: The recount3 Python package for programmatic access to uniformly processed RNA-seq data
Source: bioRxiv. 2026 Jun 19:2026.06.17.732943. Preprint. [Version 1] doi: 10.64898/2026.06.17.732943 (PMC13308058; doi:10.64898/2026.06.17.732943)
Supplement: 1 [file NIHPP2026.06.17.732943v1-supplement-1.pdf]

bioRxiv preprint doi: https://doi.org/10.64898/2026.06.17.732943; this version posted June 20, 2026. The copyright holder for this preprint (which was not certified by peer review) is the author/funder, who has granted bioRxiv a license to display the preprint in perpetuity. It is made available under a CC-BY 4.0 International license.

Supplemental Material
for

The recount3 Python package for programmatic access to uniformly processed RNA-seq data

Alexander Alsalihi[1], Robert M. Flight [1,2,3], and Hunter N. B. Moseley[1,2,3,4,5,*]

[1] Markey Cancer Center, University of Kentucky, Lexington, KY 40536, USA;
[2] Department of Molecular & Cellular Biochemistry, University of Kentucky, Lexington, KY 40536, USA
[3] Superfund Research Center, University of Kentucky, Lexington, KY 40536, USA
[4] Institute for Biomedical Informatics, University of Kentucky, Lexington, KY 40536, USA
[5] Department of Toxicology and Cancer Biology, University of Kentucky, Lexington, KY 40536, USA
* Correspondence: hunter.moseley@uky.edu

bioRxiv preprint doi: https://doi.org/10.64898/2026.06.17.732943; this version posted June 20, 2026. The copyright holder for this preprint (which was not certified by peer review) is the author/funder, who has granted bioRxiv a license to display the preprint in perpetuity. It is made available under a CC-BY 4.0 International license.

Supplemental Table 1. URL templates for downloading recount3 files.

Resource type	URL template (relative to RECOUNT3_URL)	Example
annotations	{organism}/annotations/{genomic_unit}_sums/{organism}.{genomic_unit}_sums.{annotation_extension}.gtf.gz	human/annotations/gene_sums/human.gene_sums.G026.gtf.gz
count_files_gene_or_exon	{organism}/data_sources/{data_source}/{genomic_unit}_sums/{project_shard}/{project}/{data_source}.{genomic_unit}_sums.{project}.{annotation_extension}.gz	human/data_sources/sra/gene_sums/15/SRP009615/sra.gene_sums.SRP009615.G026.gz
count_files_junctions	{organism}/data_sources/{data_source}/junctions/{project_shard}/{project}/{data_source}.junctions.{project}.{junction_type}.{junction_extension}.gz	human/data_sources/sra/junctions/15/SRP009615/sra.junctions.SRP009615.ALL.MM.gz
metadata_files	{organism}/data_sources/{data_source}/metadata/{project_shard}/{project}/{data_source}.{table_name}.{project}.MD.gz	human/data_sources/sra/metadata/15/SRP009615/sra.recount_qc.SRP009615.MD.gz
bigwig_files (SRA / TCGA)	{organism}/data_sources/{data_source}/base_sums/{project_shard}/{project}/{sample_shard}/{data_source}.base_sums.{project}_{sample}.ALL.bw	human/data_sources/sra/base_sums/15/SRP009615/77/sra.base_sums.SRP009615_SRR387777.ALL.bw
bigwig_files (GTEx)	{organism}/data_sources/gtex/base_sums/{project_shard}/{project}/{sample_shard}/gtex.base_sums.{project}_{sample}.ALL.bw	human/data_sources/gtex/base_sums/UE/ADIPOSE_TISSUE/Z7/gtex.base_sums.ADIPOSE_TISSUE_GTEX-1117F-0226-SM-5GZZ7.1.ALL.bw

bioRxiv preprint doi: https://doi.org/10.64898/2026.06.17.732943; this version posted June 20, 2026. The copyright holder for this preprint (which was not certified by peer review) is the author/funder, who has granted bioRxiv a license to display the preprint in perpetuity. It is made available under a CC-BY 4.0 International license.

data_sources	{organism}/homes_index	human/homes_index
data_source_metadata	{organism}/data_sources/{data_source}/metadata/{data_source}.recount_project.MD.gz	human/data_sources/sra/metadata/sra.recount_project.MD.gz

Path-helper notation used:

- {project_shard}: the last two characters of the project identifier (e.g. SRP009615 yields 15; ADIPOSE_TISSUE yields UE). Implemented by recount3._descriptions._project_shard.
- {sample_shard}: a two-character sample shard subdirectory whose offset depends on the data source. For SRA and TCGA it is the last two characters of the sample identifier (e.g. SRR387777 yields 77). For GTEx it is the two characters at positions [-4:-2] of the sample identifier (e.g. GTEX-1117F-0226-SM-5GZZ7.1 yields Z7). Implemented by recount3._descriptions._sample_shard.
